# Health-related quality of life, and its determinants, among older people in rural Vietnam

**DOI:** 10.1186/1471-2458-10-549

**Published:** 2010-09-11

**Authors:** Le V Hoi, Nguyen TK Chuc, Lars Lindholm

**Affiliations:** 1 Centre for Global Health Research, Department of Public Health and Clinical Medicine, Umea University, S-901 85, Umea City, Sweden; 2 Faculty of Public Health, Hanoi Medical University, 01, Ton That Tung Str., Hanoi City, Vietnam; 3 Ageing and Living Conditions Programme, Centre for Population Studies, Umeå University, S-901 85, Umea City, Sweden

## Abstract

**Background:**

The proportion of people in Vietnam aged 60 and above has increased rapidly in recent decades. However, there is a lack of evidence, particularly in rural settings, on their health-related quality of life (HRQoL) within the context of socioeconomic changes and health-sector reform in the country. This study assesses the level and determinants of HRQoL in a rural district in order to provide evidence for designing and implementing appropriate health policies.

**Methods:**

In 2007, 2,873 people aged 60+ living in 2,240 households randomly selected from the FilaBavi demographic surveillance site (DSS) were interviewed using a generic EQ-5D questionnaire to assess their HRQoL. Socioeconomic characteristics of the people and their households were extracted from the DSS's re-census that year, and the EQ-5D index was calculated based on the time trade-off tariff. Multilevel-multivariate linear regression analysis was performed to measure the affect of socioeconomic factors on HRQoL.

**Results:**

The EQ-5D index at old age was found to be 0.876 (95%CI: 0.870-0.882). Age between 60-69 or 70-79 years, position as household head, working until old age, literacy, and belonging to better wealth quintiles are determinants of higher HRQoL. Ageing has a primary influence on the deterioration of HRQoL at older ages, mainly due to reduction in physical rather than mental functions. Educational disparity in HRQoL is low, and exists mostly between basic and higher levels of education. Being a household head and working at old age are advantageous for attaining better quality of life in physical rather than psychological terms. Economic conditions affect HRQoL through sensory rather than physical utilities. Long-term living conditions more likely affect HRQoL than short-term economic conditions.

**Conclusions:**

HRQoL at old age is at a high level, and varies substantially according to socioeconomic factors. Its determinants should be addressed in social and health policies designed to improve health of older people, especially among the most vulnerable groups.

## Background

In recent decades Vietnam has experienced multiple transitions in terms of demographic, socioeconomic and epidemiological changes [1]. This demographic transition is typically characterised by a rapid ageing process with declines in both fertility and mortality. The transition from a central planning economy to a market economy has resulted in strong growth in gross domestic product (GDP) and dramatic reduction of poverty, but with wider inequality in income between regions and social groups. The epidemiological pattern is shifting from a predominance of communicable diseases to the emergence of non-communicable diseases. Within such a context, the population of older people in the country has increased dramatically in size. In particular, the proportion of people aged 60 and above within the general population grew from 6.7% in 1979 to 8.1% in 1999 [2] and up to 9.2% in 2006 [3], with this proportion set to double by 2025 and then double again by 2050 [4].

Vietnamese life expectancy at birth increased from 66 years in 1990 to 72 years in 2006 [5,6], and is projected to increase to 77.1 years and 80.3 years by 2025 and 2050, respectively [4]. There is evidence [1] that life expectancy at age 60 in a rural area increased by approximately one year from 1999-2002 to 2003-2006 but decreased amongst the most vulnerable groups. There is also a wide gap in life expectancy according to household poverty status and living arrangements, with the poverty status gap wider over the two periods [1]. Although greater life expectancy at old age is an indicator of successful ageing [7], it also means that more older people are suffering from chronic diseases [8].

In 2007 the total population of the country reached 85 million, with 72.6% residing in rural areas [9]. The majority of older people (72.9%) live in these areas [10], and are more disadvantaged than the urban elderly in terms of educational attainment, housing quality, access to media [11], and poverty status [12]. Older people, especially in rural areas, are more likely to rely on domestic sources of economic support than the social security system [13]. There is also an increasing trend of temporary migration among the young labour force from rural to urban areas because of better employment opportunities [14], which leaves more older people living on their own with less physical and emotional support from family members [15].

Since 1989, Vietnam has experienced reform within the health sector, initiated with the introduction of user fees for public services and the development of a private sector. Consequently, household health expenditure now predominantly consists of out-of-pocket payments, accounting for 67% in 2005 [16]. Although efforts have been made by the government to improve access to health care, disparities in health and health care are getting wider between socioeconomic groups, as well as between rural and urban areas. In particular, older people in rural areas have less access to health care than those in urban areas [17].

Quality of life is a subjective and multidimensional concept, which has been defined as "dynamic interactions between the external conditions of an individual's life and the internal perceptions of those conditions" [18]. The concept encompasses a wide range of aspects in human life, including physical, mental, social and spiritual functions, and environmental and material coordinates [19,20]. The perceptions depend on the context of the culture and value systems in relation to individual goals, expectations, standards and concerns [21]. Health-related quality of life (HRQoL) is increasingly used in health care research, particularly for informing patient management, policy decisions and resource allocation [18,22]. As general health and functional status are important dimensions, this domain is particularly suitable for evaluating quality of life among older people [20].

Quality of life and its health-related domains have a wide range of determinants, with socio-demographic factors and economic status of particular importance [23]. HRQoL and its determinants at old age is well documented in the developed world, but has been explored to a limited degree in developing countries [24], with little currently known about HRQoL at old age in Vietnam. Therefore, in order to provide evidence for designing health and social policies for elderly care in Vietnam, this study aims to estimate self-reported HRQoL and its socioeconomic determinants in a rural setting.

## Methods

### Study setting and the FilaBavi surveillance system

The study was conducted in 2007 within the longitudinal demographic and health surveillance system of FilaBavi [25]. This field site operates in the rural Bavi district of Vietnam, and covers an area of 410 km2, including lowland, highland and mountainous areas, of which 30% is used for agriculture and 17% is forest. In 2007 its population was 262,763 people. Among adults over 20 years of age, the majority had completed primary/secondary school (65% for males, 72% for females) and high school/higher education (34% and 23%, respectively) with the rest being illiterate. Two-thirds of the population were farmers (39% for males, 57% for females) and other workers (31% and 9%, respectively) and the remainder were business people, students, government staff, retired persons and others.

The surveillance system of FilaBavi consists of a representative sample of 67 out of 352 clusters in the district, selected randomly with a probability proportional to population size in each cluster since 1999. A cluster was defined as an administrative unit, usually a village. If a village was too large it could be divided into two clusters. On average, there were 600-700 inhabitants in each cluster. Initially, 11,089 households and 51,024 inhabitants were included for surveillance. In 2007, 53,927 individuals were followed up by FilaBavi, accounting for approximately 20.5% of the total district population. People aged 60 and over represented 11.5% of the total population followed up by FilaBavi at the mid-year point in 2007.

### Study design, sampling and sample size

A sample size of approximately 600 people in a population-based survey is required to detect an improvement at a small change of 0.02 in the EQ-5D index with an effect size (odds ratio) of 0.80 [26]. In the present study a sample size of 2,760 older people was required, having been adjusted for a design effect of 2 for cluster sampling of FilaBavi, which was then doubled for the robustness of multivariate analysis and further accounts for a non-response rate of 15%. This figure is approximately equal to 50% of all people aged 60+ in the FilaBavi sampling frame.

Subsequently, 50% of households with older people followed up by FilaBavi were randomly selected for a household cross-sectional survey, amounting to 2,255 households with 2,968 people. During the survey period from July to October 2007, 166 households were excluded due to absence of their older people, however each of these cases were then replaced with the nearest unselected household with older people. In total, 2,240 households with 2,873 older people were included in the study.

### Variable measurement and data collection

The EQ-5D questionnaire used for assessment of HRQoL has been developed by the EuroQoL Group since 1987 [27]. This instrument defines the state of general health across five dimensions (mobility, self-care, usual activities, pain/discomfort and anxiety/depression) and at three levels (no problems, some/moderate problems, severe problems). The combination of these categories theoretically results in 243 unique health states and provides an estimate of a health summary score - the EQ-5D index - on a scale where 1 is full health and 0 is deceased. The tool has been standardised and widely used in clinical and population studies in different countries [28]. As the simplest and most popular instrument for measuring HRQoL, it is feasible to apply it to a large and low literate population [29]. It is also practical for measuring HRQoL at old age [30].

To date, there are 100 official language versions of EQ-5D questionnaire, including Vietnamese, which is the version applied in this study. EQ-5D valuation sets can be used across countries, especially where one does not exist [31]. However, specific dimensions of national culture, such as power distance, individualism, masculinity and uncertainty avoidance are potential factors in providing insight into EQ-5D value set coefficients for different countries [32]. Among countries with available population-based EQ-5D preferences, South Korea have close scores with Vietnam in the most of such cultural dimensions [32]. A preference set for calculating EQ-5D indices is lacking for Vietnam, the time trade-off valuation set from South Korea [33] was used in this study.

Variables of economic status of households, including land area, structural components of housing, assets, sanitation conditions, income, expenditure and debt, were extracted from the mid-2007 FilaBavi re-census dataset. Structural components of houses were types of roof, floor and wall according to different levels of permanent or temporary materials. Assets were classified according to certain categories, such as furniture, communication and electricity equipment, types of vehicles, agricultural machines, cattle and others. These items were classified as "present or not", regardless of their quantity and quality. Sanitation conditions were assessed in terms of sources of water for drinking and cooking, type of latrine and presence of a bathroom. All types of income (from agriculture, breeding, forestry and other sources) were recorded to provide the total income of a given household. The sum of daily food expenditure was multiplied by 30 days and added to the sum of other monthly expenditure to estimate total monthly household expenditure. Monthly income and expenditure were then divided by household size to generate "per capita" variables.

Using structured questionnaires, face-to-face interviews were performed with all old people at each of the houses by 52 trained field personnel from FilaBavi. These included questions from the EQ-5D, plus others on individual and household characteristics of older people, such as date of birth, sex, education, marital status, household head status, status of living with spouse, and working status (working in own rice fields etc. or not working). Six field supervisors reviewed all completed questionnaires and randomly selected 5% for re-interviews. All questionnaires with missing or irrelevant values were returned to the field personnel for checking and completion following re-visits to the corresponding households. Double data-entry using EpiData 3.1 was performed to check inconsistent values of all variables, and then correction of data-entry errors were made based on actual values from the completed questionnaires.

### Statistical analysis

The datasets from the present survey and re-census were linked and analysed using STATA 10. Household wealth index was calculated using economic data from the re-census. Classification of household wealth quintile was based on their hierarchies among all households of FilaBavi. Household poverty status was classified using the national poverty line for rural areas, based on monthly per capita income being equal to VND 200,000 (US$ 12.5) for 2006-2010 [34].

Percentages of older people by socioeconomic group and level of EQ-5D were calculated together with their corresponding 95% confidence intervals. Average values of HRQoL by socioeconomic group were estimated together with their corresponding 95% confidence intervals. Statistically significant differences between the percentages or the average values were identified by comparing their corresponding 95% confidence intervals.

Multilevel-multivariate analyses were performed to measure the effect of socioeconomic factors on HRQoL index to a continuous scale using linear regression. Being female, ages of 80 years and above, illiteracy, widowed status, living without spouse, position as household member, not working until old age, belonged to the poorest quintile, living above the national poverty line are references for their counterparts in the analyses. A backward stepwise procedure with a p-remove of 5% was applied to identify significant factors remaining in the final multivariate model. Random effects of clusters and households were further examined in the multilevel-multivariate analysis.

### Ethical considerations

Ethical approval for the demographic surveillance system of FilaBavi, including data collection on socioeconomic statistics, was given by the Research Ethics Committee at Umeå University, Sweden (reference number 02-420). The present study was also approved by the Research Ethics Committee at Hanoi Medical University.

## Results

The socioeconomic characteristics of the study participants are summarised in Table 1. The percentage of females is almost double that of males. The majority of older people are aged 60-69 and 70-79 years. People aged 80+ account for just over one-fifth of the study population and those aged 85+ and 90+ account for 9.2% and 2.7%, respectively. Almost half had completed primary school or higher, and the illiteracy rate was 18%. Just over two-thirds are widowed and one-third are still living with their spouse, which is equal to just over half of the married elderly. Two-fifths are still working. The proportion of people living in households in the middle to richest wealth quintiles is higher than those belonging to the poorer or poorest quintiles. Approximately 15% of the study population live under the national poverty line.

**Table 1:** Distribution of older people by demographic and socioeconomic groups

Variables	n	%	95%CI
*Overall*			
60+	2,873	100.0	-
65+	2,275	79.2	77.7 - 80.7
70+	1,665	58.0	56.2 - 59.8
75+	1,096	38.2	36.4 - 39.9
80+	589	20.5	19.0 - 22.0
85+	263	9.2	8.1 - 10.2

*Age groups*			
60-69	1,208	42.0	40.2 - 43.9
70-79	1,076	37.4	35.7 - 39.2
80-89	513	17.9	16.5 - 19.3
90+	76	2.7	2.1 - 3.2

*Gender*			
Male	1,056	36.8	35.0 - 38.5
Female	1,816	63.2	61.5 - 65.0

*Education*			
High school and higher	220	7.7	6.7 - 8.6
Primary/secondary school	1,122	39.1	35.5 - 37.0
Read and write only	1,012	35.2	37.3 - 40.9
Illiterate	518	18.0	16.6 - 19.4

*Marital status*			
Married	1,569	54.8	33.0 - 56.6
Widowed	1,225	42.8	41.0 - 44.6
Separated, divorced, single	70	2.4	1.9 - 3.0

*Living with spouse*			
Yes	876	30.5	28.8 - 32.2
No	1,994	69.5	67.8 - 71.1

*Household head*			
Yes	1,493	52.1	50.2 - 53.9
No	1,374	47.9	46.1 - 49.7

*Working status*			
Yes	1,160	40.4	38.6 - 42.2
No	1,713	59.6	57.8 - 61.4

*Wealth quintiles*			
Richest	603	21.0	19.5 - 22.5
Richer	639	22.2	20.7 - 23.8
Middle	637	22.2	20.7 - 23.7
Poorer	496	17.3	15.9 - 18.7
Poorest	498	17.3	16.0 - 18.7

*National poverty line*			
Above	2,445	85.1	83.3 - 86.4
Below	428	14.9	13.6 - 16.2

### Distribution of the elderly by EQ-5D level

Table 2 describes the distribution of the elderly according to EQ-5D dimensions and levels. Across the five dimensions, pain/discomfort is the most reported problem, and is 2-4 times more frequently reported than the other dimensions. The percentage of people reporting problems within the self-care dimension is lowest, whilst those reporting problems in the dimensions of mobility, usual activities and anxiety/depression are almost equal, but approximately 1.5-2 times higher than in the self-care dimension. The percentage of people reporting moderate problems is highest in the pain/discomfort dimension, and is 3-5 times higher than in the other dimensions. The percentage of those reporting severe problems is highest in the usual activity dimension, and is 2-3 times higher than in the others.

**Table 2:** Percentage of older people by EQ-5D levels and dimensions

Problems		No			Some/moderate			Severe
**Dimensions**	**%**	**95%CI**	**%**		**95%CI**	**%**		**95%CI**

*Mobility*	84.3	83.0 - 85.6	14.3		13.0 - 15.6	1.4		1.0 - 1.8
*Self-care*	90.0	88.9 - 91.1	7.7		6.8 - 8.7	2.3		1.7 - 2.8
*Usual activities*	80.7	79.3 - 82.2	14.7		13.4 - 16.0	4.6		3.8 - 5.3
*Pain/discomfort*	61.3	59.5 - 63.1	36.1		34.3 - 37.8	2.7		2.1 - 3.2
*Anxiety/depression*	81.9	80.5 - 83.3	16.0		14.7 - 17.3	2.1		1.6 - 2.7

Tables A1-5 (see Additional file 1) present the percentage presence of problems in each dimension. The percentage of some/moderate problems is higher by approximately 30-60% among females than males in all dimensions, including borderline significance in the anxiety/depression dimension. The gender-gap in suffering some/moderate problems is highest in the self-care dimension and lowest in the anxiety/depression dimension. A significant difference in the percentage of severe problems by sexes only exists in the anxiety/depression dimension, where severe problems were reported more among females than males. The percentage of females reporting severe problems is highest in the usual activity dimension, while there is no significant difference in this proportion across the other dimensions.

The proportion of the study population reporting moderate problems increases with age, by at least 50% in the mobility and self-care dimensions, and at least 25% in the usual activity and pain/discomfort dimensions. The proportion reporting severe problems in the two oldest groups is at least 3-5 times that of the younger groups in each dimension. However, there is no significant difference in the percentage reporting problems in the anxiety/depression dimension across the different age groups.

The proportion of people reporting no problems is greater among those with higher levels of education across all dimensions, except for insignificant differences between those who have completed primary/secondary school and those who have completed high school or higher in some dimensions. In the self-care dimension, people with a lower level of education were more likely to report severe problems, particularly amongst those educated to primary/secondary level and below. A higher proportion of illiterate people reported severe problems in the usual activity dimension than those who are literate, and similarly than those with primary education in the mobility, self-care and anxiety/depression dimensions. In the pain/discomfort and anxiety/depression dimensions, those who had completed high school and higher education have the lowest proportion reporting moderate problems than any other socioeconomic status.

Across all dimensions, married people were more likely to report no problems and less likely to report some/moderate problems than those who were widowed. In the self-care, usual activities and anxiety/depression dimensions, severe problems are reported more frequently among married people than those who are widowed. Anxiety/depression at moderate level is mostly reported among those who are separated, divorced or single than those with any other socioeconomic status. People living with a spouse were more likely to report no problems and less likely to report some/moderate problems than their counterparts in all dimensions.

In all but the anxiety/depression dimension, household heads were more likely to report no problems and less likely to report some/moderate problems than those who were not household heads. Severe problems are less frequently reported among household heads than non-household heads in the mobility, self-care and usual activity dimensions.

In all dimensions, those who are still working were more likely to report no problems and less likely to report some/moderate problems than their counterparts. A higher proportion of those people who still work reported severe problems than their counterparts, except in the anxiety/depression dimension.

In terms of wealth quintiles, the percentage reporting some/moderate problems was lower among the richest than those in the poorer and poorest quintiles for the pain/discomfort dimension. In the anxiety/depression dimension, the proportion reporting some/moderate problems was lowest among the richest quintile and highest among the poorest quintile. Severe problems are more frequent among the poorest than the richer and richest quintiles for the anxiety/depression dimension, and are most frequent among the poorest than any other socioeconomic status. Moderate anxiety/depression among the poorest is only lower than that among separated, divorced or single people. In the pain/discomfort dimension, the percentage reporting severe problems is only lower among the poorest quintile than among the oldest people, yet not significantly so.

People living above the national poverty line were more likely to report no problems and less likely to report some/moderate problems than their counterparts in all dimensions, excluding the usual activity dimension. In each dimension except for pain/discomfort, the proportion reporting moderate problems is only lower among people living under the poverty line than among the oldest group and illiterate people, either significantly or not. In the anxiety/depression dimension, the proportion reporting severe problems among people living under the poverty line makes it one of the top three socioeconomic status categories also reporting severe problems, together with the poorest wealth quintile and the illiterate elderly.

### Socioeconomic variations of the EQ-5D index at old age

The mean EQ-5D indices by socioeconomic groups of older people are presented in Table 3. Males have a higher index than females, and the indices decrease with age. The indices increase with level of education, except between the primary/secondary education and higher levels. Married people have higher indices than those who are separated, divorced or single, and the indices for people living with their spouse, household heads and those who still work are all higher than for their respective counterparts. People belonging to the middle to richest quintiles have higher indices than those in the poorest quintile, and the index among people living beyond the national poverty line is higher than those living below, but only with borderline significance.

**Table 3:** Estimates of EQ-5D index among older people by socioeconomic group

*Variables*	*EQ-5D index*	*95%CI*
*Overall*		
60+	0.876	0.870 - 0.882
65+	0.865	0.858 - 0.872
70+	0.853	0.844 - 0.861
75+	0.833	0.821 - 0.844
80+	0.796	0.778 - 0.815
85+	0.742	0.709 - 0.775

*Age groups*		
60-69	0.908	0.902 - 0.914
70-79	0.884	0.876 - 0.892
80-89	0.815	0.797 - 0.834
90+	0.668	0.602 - 0.736

*Gender*		
Male	0.893	0.885 - 0.901
Female	0.866	0.858 - 0.873

*Education*		
High school and higher	0.908	0.891 - 0.924
Primary/secondary school	0.901	0.894 - 0.908
Read and write only	0.870	0.860 - 0.880
Illiterate	0.819	0.801 - 0.837

*Marital status*		
Married	0.897	0.891 - 0.904
Widowed	0.873	0.843 - 0.904
Separated, divorced, single	0.848	0.838 - 0.858

*Living with spouse*		
Yes	0.894	0.885 - 0.903
No	0.860	0.861 - 0.875

*Household head*		
Yes	0.894	0.888 - 0.900
No	0.856	0.846 - 0.865

*Working status*		
Yes	0.917	0.913 - 0.922
No	0.848	0.838 - 0.857

*Wealth quintiles*		
Richest	0.881	0.867 - 0.893
Richer	0.883	0.872 - 0.894
Middle	0.881	0.870 - 0.892
Poorer	0.872	0.858 - 0.885
Poorest	0.859	0.844 - 0.874

*National poverty line*		
Above	0.879	0.873 - 0.885
Below	0.858	0.842 - 0.873

The affect of socioeconomic factors on older people's EQ-5D index obtained from multilevel-multivariate regression analyses using linear models is presented in Tables 4. Ages of 60-69 and 70-79 years, literacy, belonged to middle to richest quintiles, household head status and working until old age are good indicators of having better EQ-5D indices. Although there is a significant difference in the EQ-5D between marital statuses, living with or without a spouse and above or below the national poverty line in the simple analysis, these factors are no longer present in the final model of the hierarchy analysis. Random effects of clusters or households exist at the same levels when comparing each of the model.

**Table 4:** Affect of socioeconomic factors on EQ-5D index among older people from multilevel-multivariate linear regression analysis

*Terms*	*Coefficients*	*P*	*95%CI of Coefficients*
*Fixed effects*			
Aged 60-69	0.067	<0.001	0.049 - 0.084
Aged 70-79	0.064	<0.001	0.048 - 0.079
High school or higher	0.032	0.018	0.006 - 0.058
Secondary/primary school	0.025	0.006	0.007 - 0.043
Read and write only	0.019	0.020	0.003 - 0.036
Household head	0.017	0.003	0.006 - 0.029
Working	0.053	<0.001	0.041 - 0.066
Richest quintile	0.024	0.004	0.008 - 0.040
Richer quintile	0.021	0.007	0.006 - 0.036
Middle quintile	0.016	0.033	0.001 - 0.031
Constant	0.759	<0.001	0.740 - 0.777

*Random effects*			
Cluster (*sd*)	0.037		0.029 - 0.047
Household (*sd*)	0.025		0.008 - 0.075
Residual (*sd*)	0.140		0.135 - 0.146

References: aged 80+, illiteracy, household member; not working, poorest quintile

## Discussion

HRQoL at old age in rural areas reaches an average level of 0.876 and varies substantially according to socioeconomic determinants, particularly for age group, educational level, marital status, household head status, working status and household wealth quintiles. To the best of our knowledge, there are only a few population-based studies of HRQoL at old age in Vietnam. Since the present study is the first to apply the EQ-5D to a general population of older people, there are no comparable figures from previous studies. Our finding that HRQoL varies between socioeconomic status is consistent with that of previous population-based studies using the EQ-5D in other countries [35-39].

### Statistical and clinical significance of variations in HRQoL

Although there is currently no explicit agreement on a clinically important difference for the EQ-5D, a range from 0.033 [40] to 0.074 [41] for minimally important differences between socioeconomic groups and groups with particular clinical conditions has been advocated [35]. The shift in age groups and working status that constitute changes in the EQ-5D index of at least 0.064 and 0.053 respectively, are within this range. Variations of the index according to other determinants, particularly household wealth quintiles (0.016-0.024), education levels (0.019-0.032) and household head status (0.017), are lower than this range, and thus not clinically significant.

Therefore, the clinically significant socioeconomic factors should be considered in evaluations of the effectiveness of clinical or public health interventions for improving HRQoL at old age, especially controlling for the influence of age. Applicable to current Vietnamese policies on social and health care for older people, age can be a good indicator for setting target groups of older people involved in social and health care interventions for improving HRQoL in a context with limited resources.

### Primary affect of ageing in deterioration of HRQoL

Measurement of HRQoL using the EQ-5D is more oriented towards functional dimensions, which evidently deteriorate at older ages [35,42,43]. The remarkable decrease in HRQoL at older ages in the present study supports the findings of previous studies. Variation in the EQ-5D index by age group is greater after simultaneously adjusting for the effects of other socioeconomic determinants, and is greater when compared with those of the other factors. These findings confirm the primary affect of age in reducing HRQoL among the rural elderly.

Deterioration in HRQoL by age group is remarkable in most of the dimensions, but not for anxiety/depression. We surmise that this decrease with age is mainly contributable to the decline in physical rather than mental functions. Although varying insignificantly by age group, the proportion reporting problems within the mental dimension are equal to or higher when compared with those in the dimensions of physical functions. Therefore, interventions for improving HRQoL at old age should address both problems of deteriorating physical functions at the older ages and the need to reduce the same level of mental problems at all ages.

In terms of dimensions of physical functions, the proportion reporting problems - especially at the severe level - is much higher among people aged 80-89 and 90+ than among the younger groups and any other socioeconomic group, which suggests that this group of oldest people are the most vulnerable. In an attempt to improve the health status of the elderly, all Vietnamese people aged 85+ have been granted free health insurance since 2007, as an extension to a policy introduced in 2003 under limited public resources which applied only to those aged 90+ [16]. Using evidence from the present study, the earlier policy would reach only approximately 3% of older people who are 90+ in the rural setting. The age extension will cover more, but still up to only half of the most vulnerable group. Therefore, current and future social and health policies for improving HRQoL at old age should be extended further to cover the most vulnerable groups.

### Evidence on gender disparity in HRQoL at old age

Variation in HRQoL by gender has been observed at different levels in other countries, and it has been found to be more likely that the gender difference is statistically insignificant [43], or that females are disadvantaged in the EQ-5D index than males [35,36,39,44,45]. Gender disparity has been argued to be greater because EQ-5D may not capture intermittent but more common symptoms among women, such as migraine or major depression [46].

In the present study, the gender gap is greatest amongst those reporting severe problems in the usual activity dimension, and those reporting moderate problems in the self-care dimension. This suggests that the interventions for reducing gender gaps in HRQoL at old age should pay greatest attention to these physical dimensions.

### Affect of education on HRQoL at old age

Education has been widely identified as a determinant of health outcomes as it shapes occupational opportunities and earning potential [47], which consequently affect living standards and health care. Education also provides basic knowledge and life skills to get better access to information and resources to promote health during the whole lifespan [48]. As HRQoL is a subjective health outcome, people with different levels of educational attainment can perceive its dimensions differently, particularly with regard to psychological functions. Previous studies have found that people with higher levels of educational attainment usually have higher EQ-5D indices [35,36,43,44].

In the present study, literate older people have a significantly higher index with a difference of 0.019-0.032. This reconfirms the affect - albeit low - of basic education on HRQoL at old age, and relates to the fact that educational gaps in the dimensions mainly exist between the lower levels of education and among moderate levels of problems. Successful implementation in Vietnam and other developing countries of the so-called "Education for All" strategy may have a long-term positive impact on the educational gap at old age in the future.

### Influence of household head status on HRQoL

It has been observed in the same rural area that older people who are household heads have a better general health status than those who are household members [1]. This reflects the important role of household decision-making in improving health status among the elderly, particularly relating to household health expenditure and food consumption. The present study provides additional evidence that older people who are household heads have better EQ-5D indices than those who are household members.

Furthermore, in all dimensions except anxiety/depression, the proportion of people reporting problems was lower among household heads than household members. This suggests that being a household head has more efficacy in physical rather than psychological terms.

### Role of working status in the mental dimension of HRQoL

A smaller proportion of older, working people report problems than those who are not working. Moreover, under almost all dimensions, the proportion of working people who report problems is lower compared with any other socioeconomic status. Consequently, working people have a much higher EQ-5D index than their counterparts.

Variation by working status is equal to that by age group, and is highest when compared with those caused by the other determinants considered in this study. It is also notable that the proportion reporting problems by working status are equal at all levels for the anxiety/depression dimension, which suggests that working status affects HRQoL through physical rather than mental dimensions.

### Disparities in HRQoL caused by economic conditions

EQ-5D indices are lower among the poorer and poorest than among other wealth quintiles. However, significant differences in the proportion reporting problems between the poorer or poorest and other wealth quintiles only exist in the pain/discomfort and anxiety/depression dimensions. This indicates that the poorest and poorer are the most vulnerable in terms of household living conditions and low HRQoL at old age, and that these conditions affect HRQoL through mental rather than physical dimensions.

On the other hand, variations in the EQ-5D index according to position on the national poverty line are not significant once adjusted for other factors in the hierarchy analyses. This finding suggests that HRQoL at old age is more likely affected by long-term economic conditions than short-term conditions, as measured by per capita household income.

### Cross-country comparison of HRQoL at old age

EQ-5D indices among 60-69- and 70-79-year-old Vietnamese study participants are higher than those for the same age groups among Swedes measured in 1998 (0.80 with SE = 0.010 and 0.79 with SE = 0.012, respectively) [36], and Americans measured in 2000-2002 (0.823 with SE = 0.003 and 0.790 with SE = 0.004, respectively) [49]. This could be due to the fact that HRQoL in rural Vietnam reaches beyond recently set EQ-5D levels in these developed countries. There is evidence to support the improvement of objectively measured overall health status in Vietnam, however: firstly, life expectancy at birth is better in Vietnam than in other countries at the same economic development level [50]; secondly, life expectancy at old age in rural areas is equal to that of a developed country two decades ago [1].

These cross-country comparisons should be treated with caution, however, because HRQoL, assessed by the EQ-5D instrument as a subjective measurement of general health status can vary among people according to their cultural or social norms and differences in expectations regarding health. EQ-5D index scores at old ages from other Asian countries that are comparable with the figures from the current study are not available.

### Methodological issues

Certain methodological limitations should be discussed in interpreting the relationship between socioeconomic determinants and HRQoL using EQ-5D among rural people. First, although Vietnam and South Korea have relatively similar scores in many dimensions of national culture that potentially affect EQ-5D value set coefficients, it is still questionable how much value the preference from this Asian country carries for a Vietnamese population. Secondly, measurement of HRQoL using EuroQol's instrument is only useful for the day of data collection, and may not capture intermittent symptoms, especially among women.

Thirdly, household economic data, particularly expenditure and income, were extracted from re-census data and these figures usually fluctuate to some extent, especially by seasons in rural areas of Vietnam. Fourth, the proportion of older people who are illiterate or can barely read or write is still high in rural areas, and it should be considered that people with low literacy may understand the survey questions differently. Fifth, the significant relationship from this cross-sectional survey does not allow for any inferential explanation of causal pathway between HRQoL at the old age and its determinants.

## Conclusions

HRQoL at old age is at a high level, and varies substantially according to socioeconomic factors. Younger age, position as a household head, working until old age, literacy, and belonging to better wealth quintiles are all determinants of higher HRQoL among older people in this rural area of Vietnam.

Ageing has a primary influence on the deterioration of HRQoL at older ages, mainly due to reduction in physical rather than mental functions. Educational disparity in HRQoL is low, and exists mostly between basic and higher levels of education. Being a household head and working at old age are advantageous for attaining better quality of life in physical rather than psychological terms. Economic conditions affect HRQoL through sensory rather than physical utilities. Finally, long-term living conditions more likely affect HRQoL than short-term economic conditions.

Socioeconomic determinants are of importance in evaluating HRQoL at old age, and should be addressed in social and health policies designed to improve health among these vulnerable groups in the context of multiple socioeconomic transitions.

## Competing interests

The authors declare that they have no competing interests.

## Authors' contributions

LVH conceived and designed the research, developed its tools, supervised data collection, analysed data and drafted the manuscript. NTKC advised on research design, supervised data collection and revised the manuscript. LL assisted during the conception phase, advised on the research design and data analysis, and revised the draft manuscript. All authors read and approved the final manuscript.

## Pre-publication history

The pre-publication history for this paper can be accessed here:

http://www.biomedcentral.com/1471-2458/10/549/prepub

## Supplementary Material

Additional file 1 **Annex**. Distribution of older people among socioeconomic groups by levels of EQ-5D Click here for file
