# Supplementary material for: Health-related quality of life, and its determinants, among older people in rural Vietnam
Source: BMC Public Health. 2010 Sep 11;10:549. doi: 10.1186/1471-2458-10-549 (PMC2944376; doi:10.1186/1471-2458-10-549)
Supplement: Additional file 1 — Annex. Distribution of older people among socioeconomic groups by levels of EQ-5D [file 1471-2458-10-549-S1.DOC]

**Annex:**

**Distribution of older people**

**among socioeconomic groups by levels of EQ-5D**

**Table A1 – Percentage of older people among socioeconomic groups**

**by levels of mobility dimension**

| *Problems* |  | | *No* | | |  |  | | *Some/moderate* | | |  |  | *Severe* | | |  |
| --- | --- | --- | --- | --- | --- | --- | --- | --- | --- | --- | --- | --- | --- | --- | --- | --- | --- |
| *Socioeconomic groups* | *%* | | | *95%CI* | | | *%* | | | *95%CI* | | | *%* | | *95%CI* | | |
| *Age groups* |  | | |  | | |  | | |  | | |  | |  | | |
| 60–69 | 91.8 | | | 90.3 – 93.4 | | | 7.8 | | | 6.3 – 9.3 | | | 0.4 | | 0.1 – 0.8 | | |
| 70–79 | 85.9 | | | 83.8 – 88.0 | | | 13.5 | | | 11.4 – 15.5 | | | 0.7 | | 0.2 – 1.1 | | |
| 80–89 | 69.2 | | | 65.2 – 73.2 | | | 26.9 | | | 23.1 – 38.8 | | | 3.9 | | 2.2 – 5.6 | | |
| 90+ | 46.1 | | | 34.6 – 57.5 | | | 43.4 | | | 32.0 – 54.8 | | | 10.5 | | 3.5 – 17.6 | | |
| *Gender* |  | | |  | | |  | | |  | | |  | |  | | |
| Male | 87.3 | | | 85.3 – 89.3 | | | 11.7 | | | 9.8 – 13.7 | | | 1.0 | | 0.4 – 1.5 | | |
| Female | 82.6 | | | 80.9 – 84.3 | | | 15.8 | | | 14.1 – 17.4 | | | 1.7 | | 1.1 – 2.2 | | |
| *Education* |  | | |  | | |  | | |  | | |  | |  | | |
| High school and higher | 90.5 | | | 86.5 – 94.4 | | | 8.6 | | | 1.9 – 12.4 | | | 0.9 | | -0.4 – 2.2 | | |
| Primary/secondary school | 89.7 | | | 87.9 – 91.4 | | | 9.7 | | | 0.9 – 11.5 | | | 0.6 | | 0.2 – 1.1 | | |
| Read and write only | 83.0 | | | 80.7 – 85.3 | | | 15.6 | | | 13.4 – 17.9 | | | 1.4 | | 0.7 – 2.1 | | |
| Illiterate | 72.8 | | | 68.9 – 76.6 | | | 23.9 | | | 20.3 – 27.6 | | | 3.3 | | 1.7 – 4.8 | | |
| *Marital status* |  | | |  | | |  | | |  | | |  | |  | | |
| Married | 89.1 | | | 87.6 – 90.6 | | | 10.0 | | | 8.5 – 11.5 | | | 0.9 | | 0.4 – 1.4 | | |
| Widowed | 78.3 | | | 76.0 – 80.6 | | | 19.6 | | | 17.4 – 21.8 | | | 2.1 | | 1.3 – 2.9 | | |
| Separated, divorced, single | 81.4 | | | 72.1 – 90.8 | | | 18.6 | | | 9.2 – 27.9 | | | – | | – | | |
| *Living with spouse* |  | | |  | | |  | | |  | | |  | |  | | |
| Yes | 88.2 | | | 86.1 – 90.4 | | | 11.1 | | | 9.0 – 13.2 | | | 0.7 | | 0.1 – 1.2 | | |
| No | 82.6 | | | 81.0 – 84.3 | | | 15.6 | | | 14.1 – 17.2 | | | 1.7 | | 1.1 – 2.3 | | |
| *Household head* | |  | | |  | | |  | | |  | |  | | |  | |
| Yes | | 87.6 | | | 85.9 – 89.3 | | | 11.9 | | | 10.2 – 13.5 | | 0.5 | | | 0.2 – 0.9 | |
| No | | 80.7 | | | 78.6 – 82.8 | | | 17.0 | | | 15.0 – 18.9 | | 2.3 | | | 1.5 – 3.1 | |
| *Working status* | |  | | |  | | |  | | |  | |  | | |  | |
| Yes | | 94.7 | | | 93.5 – 96.0 | | | 5.2 | | | 3.9 – 6.4 | | 0.1 | | | -0.1 – 0.3 | |
| No | | 77.3 | | | 75.3 – 79.3 | | | 20.4 | | | 18.5 – 22.3 | | 2.3 | | | 1.6 – 3.0 | |
| *Wealth quintiles* | |  | | |  | | |  | | |  | |  | | |  | |
| Richest | | 85.7 | | | 82.9 – 88.5 | | | 12.1 | | | 9.5 – 14.7 | | 2.2 | | | 1.0 – 3.3 | |
| Richer | | 83.4 | | | 80.5 – 86.3 | | | 15.8 | | | 13.0 – 18.6 | | 0.8 | | | 0.1 – 1.5 | |
| Middle | | 85.6 | | | 82.8 – 88.3 | | | 13.3 | | | 10.7 – 16.0 | | 1.1 | | | 0.3 – 1.9 | |
| Poorer | | 83.3 | | | 80.0 – 86.6 | | | 15.5 | | | 12.3 – 18.7 | | 1.2 | | | 0.2 – 2.2 | |
| Poorest | | 83.3 | | | 80.0 – 86.6 | | | 14.9 | | | 11.7 – 18.0 | | 1.8 | | | 0.6 – 3.0 | |
| *National poverty line* | |  | | |  | | |  | | |  | |  | | |  | |
| Above | | 85.4 | | | 84.0 – 86.8 | | | 13.3 | | | 11.9 – 14.6 | | 1.3 | | | 0.9 – 1.8 | |
| Below | | 78.3 | | | 74.3 – 82.2 | | | 20.1 | | | 16.3 – 23.9 | | 1.6 | | | 0.4 – 2.8 | |

**Table A2 – Percentage of older people among socioeconomic groups**

**by levels of self-care dimension**

| *Problems* |  | | *No* | | |  |  | | *Some/moderate* | | |  |  | *Severe* | | |  |
| --- | --- | --- | --- | --- | --- | --- | --- | --- | --- | --- | --- | --- | --- | --- | --- | --- | --- |
| *Socioeconomic groups* | *%* | | | *95%CI* | | | *%* | | | *95%CI* | | | *%* | | *95%CI* | | |
| *Age groups* |  | | |  | | |  | | |  | | |  | |  | | |
| 60–69 | 95.7 | | | 94.5 – 96.8 | | | 3.7 | | | 2.7 – 4.8 | | | 0.6 | | 0.2 – 1.0 | | |
| 70–79 | 92.1 | | | 90.5 – 93.7 | | | 6.6 | | | 5.1 – 8.1 | | | 1.3 | | 0.6 – 2.0 | | |
| 80–89 | 78.8 | | | 75.2 – 82.3 | | | 15.6 | | | 12.5 – 18.8 | | | 5.7 | | 3.7 – 7.7 | | |
| 90+ | 45.3 | | | 33.8 – 56.9 | | | 34.7 | | | 23.6 – 45.7 | | | 20.0 | | 10.7 – 29.3 | | |
| *Gender* |  | | |  | | |  | | |  | | |  | |  | | |
| Male | 92.9 | | | 91.3 – 94.4 | | | 5.5 | | | 4.1 – 6.9 | | | 1.6 | | 0.8 – 2.4 | | |
| Female | 88.3 | | | 86.8 – 89.8 | | | 9.0 | | | 7.7 – 10.4 | | | 2.6 | | 1.9 – 3.4 | | |
| *Education* |  | | |  | | |  | | |  | | |  | |  | | |
| High school and higher | 96.8 | | | 94.5 – 99.2 | | | 2.3 | | | 0.3 – 4.3 | | | 0.9 | | -0.4 – 2.2 | | |
| Primary/secondary school | 94.6 | | | 93.2 – 95.9 | | | 4.5 | | | 3.3 – 5.8 | | | 0.9 | | 0.3 – 1.4 | | |
| Read and write only | 89.4 | | | 87.5 – 91.3 | | | 8.0 | | | 6.3 – 9.7 | | | 2.6 | | 1.6 – 3.5 | | |
| Illiterate | 78.4 | | | 74.8 – 81.9 | | | 16.4 | | | 13.2 – 19.6 | | | 5.2 | | 3.3 – 7.1 | | |
| *Marital status* |  | | |  | | |  | | |  | | |  | |  | | |
| Married | 93.7 | | | 92.5 – 94.9 | | | 5.0 | | | 4.0 – 6.1 | | | 1.3 | | 0.7 – 1.8 | | |
| Widowed | 85.4 | | | 83.4 – 87.4 | | | 11.0 | | | 9.3 – 12.8 | | | 3.6 | | 2.6 – 4.6 | | |
| Separated, divorced, single | 87.1 | | | 79.1 – 95.2 | | | 11.4 | | | 3.8 – 19.1 | | | 1.4 | | -1.4 – 4.3 | | |
| *Living with spouse* |  | | |  | | |  | | |  | | |  | |  | | |
| Yes | 93.6 | | | 92.0 – 95.2 | | | 5.0 | | | 3.6 – 6.5 | | | 1.4 | | 0.6 – 2.1 | | |
| No | 88.4 | | | 87.0 – 89.8 | | | 8.9 | | | 7.7 – 10.2 | | | 2.7 | | 2.0 – 3.4 | | |
| *Household head* | |  | | |  | | |  | | |  | |  | | |  | |
| Yes | | 93.9 | | | 92.7 – 95.1 | | | 5.3 | | | 4.2 – 6.4 | | 0.8 | | | 0.4 – 1.3 | |
| No | | 85.7 | | | 83.9 – 87.6 | | | 10.4 | | | 8.8 – 12.0 | | 3.9 | | | 2.8 – 4.9 | |
| *Working status* | |  | | |  | | |  | | |  | |  | | |  | |
| Yes | | 97.9 | | | 97.1 – 98.8 | | | 1.9 | | | 1.1 – 2.7 | | 0.2 | | | -0.1 – 0.4 | |
| No | | 84.6 | | | 82.9 – 86.3 | | | 11.7 | | | 10.2 – 13.2 | | 3.7 | | | 2.8 – 4.6 | |
| *Wealth quintiles* | |  | | |  | | |  | | |  | |  | | |  | |
| Richest | | 90.9 | | | 88.6 – 93.2 | | | 6.6 | | | 4.6 – 8.6 | | 2.5 | | | 1.2 – 3.7 | |
| Richer | | 89.7 | | | 87.3 – 92.0 | | | 8.3 | | | 6.2 – 10.4 | | 2.0 | | | 0.9 – 3.1 | |
| Middle | | 92.0 | | | 89.9 – 94.1 | | | 5.5 | | | 3.7 – 7.3 | | 2.5 | | | 1.3 – 3.7 | |
| Poorer | | 89.5 | | | 86.8 – 92.2 | | | 8.3 | | | 5.8 – 10.7 | | 2.2 | | | 0.9 – 3.5 | |
| Poorest | | 87.3 | | | 84.4 – 90.3 | | | 10.6 | | | 1.4 – 13.4 | | 2.0 | | | 0.8 – 3.2 | |
| *National poverty line* | |  | | |  | | |  | | |  | |  | | |  | |
| Above | | 90.7 | | | 89.5 – 91.8 | | | 7.1 | | | 6.1 – 8.1 | | 2.3 | | | 1.7 – 2.8 | |
| Below | | 86.2 | | | 82.9 – 89.5 | | | 11.4 | | | 8.4 – 14.5 | | 2.3 | | | 0.9 – 3.8 | |

**Table A3 – Percentage of older people among socioeconomic groups**

**by levels of usual activity dimension**

| *Problems* |  | | *No* | | |  |  | | *Some/moderate* | | |  |  | *Severe* | | |  |
| --- | --- | --- | --- | --- | --- | --- | --- | --- | --- | --- | --- | --- | --- | --- | --- | --- | --- |
| *Socioeconomic groups* | *%* | | | *95%CI* | | | *%* | | | *95%CI* | | | *%* | | *95%CI* | | |
| *Age groups* |  | | |  | | |  | | |  | | |  | |  | | |
| 60–69 | 91.2 | | | 89.6 – 92.8 | | | 7.5 | | | 6.0 – 9.0 | | | 1.2 | | 0.6 – 1.9 | | |
| 70–79 | 81.1 | | | 78.8 – 83.5 | | | 15.3 | | | 13.2 – 17.5 | | | 3.5 | | 2.4 – 4.6 | | |
| 80–89 | 62.2 | | | 58.0 – 66.4 | | | 27.5 | | | 23.6 – 31.4 | | | 10.3 | | 7.7 – 13.0 | | |
| 90+ | 32.9 | | | 22.1 – 43.7 | | | 34.2 | | | 23.3 – 45.1 | | | 32.9 | | 22.1 – 43.7 | | |
| *Gender* |  | | |  | | |  | | |  | | |  | |  | | |
| Male | 84.8 | | | 82.7 – 87.0 | | | 11.7 | | | 9.8 – 13.7 | | | 3.4 | | 2.3 – 4.5 | | |
| Female | 78.3 | | | 76.4 – 80.2 | | | 16.5 | | | 14.8 – 18.2 | | | 5.2 | | 4.2 – 6.3 | | |
| *Education* |  | | |  | | |  | | |  | | |  | |  | | |
| High school and higher | 88.2 | | | 83.9 – 92.5 | | | 10.5 | | | 6.4 – 14.5 | | | 1.4 | | -0.2 – 2.9 | | |
| Primary/secondary school | 88.1 | | | 86.2 – 90.0 | | | 9.4 | | | 7.7 – 11.1 | | | 2.6 | | 1.7 – 3.5 | | |
| Read and write only | 79.5 | | | 77.1 – 82.0 | | | 16.3 | | | 14.0 – 18.6 | | | 4.2 | | 2.9 – 5.4 | | |
| Illiterate | 63.9 | | | 59.7 – 68.0 | | | 25.1 | | | 21.4 – 28.8 | | | 11.0 | | 8.3 – 13.7 | | |
| *Marital status* |  | | |  | | |  | | |  | | |  | |  | | |
| Married | 87.0 | | | 85.3 – 88.7 | | | 10.4 | | | 8.9 – 11.9 | | | 2.6 | | 1.8 – 3.4 | | |
| Widowed | 72.6 | | | 70.1 – 75.1 | | | 20.3 | | | 18.1 – 22.6 | | | 7.1 | | 5.7 – 8.5 | | |
| Separated, divorced, single | 80.0 | | | 70.4 – 89.6 | | | 15.7 | | | 7.0 – 24.5 | | | 4.3 | | -0.6 – 9.1 | | |
| *Living with spouse* |  | | |  | | |  | | |  | | |  | |  | | |
| Yes | 84.9 | | | 82.6 – 87.3 | | | 11.9 | | | 9.7 – 14.0 | | | 3.2 | | 2.0 – 4.4 | | |
| No | 78.9 | | | 77.1 – 80.7 | | | 15.9 | | | 14.3 – 17.5 | | | 5.2 | | 4.2 – 6.1 | | |
| *Household head* | |  | | |  | | |  | | |  | |  | | |  | |
| Yes | | 85.6 | | | 83.8 – 87.4 | | | 12.2 | | | 10.5 – 13.9 | | 2.2 | | | 1.5 – 3.0 | |
| No | | 75.3 | | | 73.0 – 77.6 | | | 17.5 | | | 15.5 – 19.6 | | 7.1 | | | 5.8 – 8.5 | |
| *Working status* | |  | | |  | | |  | | |  | |  | | |  | |
| Yes | | 93.9 | | | 92.5 – 95.3 | | | 5.6 | | | 4.3 – 6.9 | | 0.5 | | | 0.1 – 0.9 | |
| No | | 71.8 | | | 69.7 – 73.9 | | | 20.9 | | | 19.0 – 22.8 | | 7.3 | | | 6.1 – 8.5 | |
| *Wealth quintiles* | |  | | |  | | |  | | |  | |  | | |  | |
| Richest | | 82.4 | | | 79.4 – 85.5 | | | 12.8 | | | 10.1 – 15.4 | | 4.8 | | | 3.1 – 6.5 | |
| Richer | | 80.3 | | | 77.2 – 83.4 | | | 15.8 | | | 13.0 – 18.6 | | 3.9 | | | 2.4 – 5.4 | |
| Middle | | 82.4 | | | 79.5 – 85.4 | | | 13.3 | | | 10.7 – 16.0 | | 4.2 | | | 2.7 – 5.8 | |
| Poorer | | 78.4 | | | 74.8 – 82.1 | | | 16.5 | | | 13.3 – 19.8 | | 5.0 | | | 3.1 – 7.0 | |
| Poorest | | 79.3 | | | 75.7 – 82.9 | | | 15.7 | | | 12.5 – 18.9 | | 5.0 | | | 3.1 – 6.9 | |
| *National poverty line* | |  | | |  | | |  | | |  | |  | | |  | |
| Above | | 81.9 | | | 80.4 – 83.4 | | | 13.5 | | | 12.1 – 14.9 | | 4.6 | | | 3.8 – 5.4 | |
| Below | | 73.8 | | | 69.7 – 78.0 | | | 21.7 | | | 17.8 – 25.7 | | 4.4 | | | 2.5 – 6.4 | |

**Table A4 – Percentage of older people among socioeconomic groups**

**by levels of pain/discomfort dimension**

| *Problems* |  | | *No* | | |  |  | | *Some/moderate* | | |  |  | *Severe* | | |  |
| --- | --- | --- | --- | --- | --- | --- | --- | --- | --- | --- | --- | --- | --- | --- | --- | --- | --- |
| *Socioeconomic groups* | *%* | | | *95%CI* | | | *%* | | | *95%CI* | | | *%* | | *95%CI* | | |
| *Age groups* |  | | |  | | |  | | |  | | |  | |  | | |
| 60–69 | 71.0 | | | 1.3 – 73.6 | | | 27.1 | | | 24.6 – 29.6 | | | 1.9 | | 1.1 – 2.7 | | |
| 70–79 | 58.8 | | | 55.9 – 61.8 | | | 38.9 | | | 36.0 – 41.9 | | | 2.2 | | 1.3 – 3.1 | | |
| 80–89 | 47.8 | | | 43.4 – 52.1 | | | 48.0 | | | 43.6 – 52.3 | | | 4.3 | | 2.5 – 6.1 | | |
| 90+ | 32.9 | | | 22.1 – 43.7 | | | 57.9 | | | 46.5 – 69.3 | | | 9.2 | | 2.6 – 15.9 | | |
| *Gender* |  | | |  | | |  | | |  | | |  | |  | | |
| Male | 69.8 | | | 67.0 – 72.6 | | | 27.9 | | | 25.2 – 30.6 | | | 2.3 | | 1.4 – 3.2 | | |
| Female | 56.3 | | | 54.0 – 58.6 | | | 40.8 | | | 38.5 – 43.1 | | | 2.9 | | 2.1 – 3.6 | | |
| *Education* |  | | |  | | |  | | |  | | |  | |  | | |
| High school and higher | 74.5 | | | 68.7 – 80.3 | | | 23.2 | | | 17.6 – 28.8 | | | 2.3 | | 0.3 – 4.3 | | |
| Primary/secondary school | 67.4 | | | 64.6 – 70.1 | | | 31.0 | | | 28.3 – 33.7 | | | 1.6 | | 0.9 – 2.3 | | |
| Read and write only | 57.6 | | | 54.6 – 60.7 | | | 39.0 | | | 36.0 – 42.0 | | | 3.4 | | 2.2 – 4.5 | | |
| Illiterate | 49.6 | | | 45.3 – 53.9 | | | 46.7 | | | 42.4 – 51.0 | | | 3.7 | | 2.0 – 5.3 | | |
| *Marital status* |  | | |  | | |  | | |  | | |  | |  | | |
| Married | 68.1 | | | 65.8 – 70.4 | | | 29.7 | | | 27.4 – 32.0 | | | 2.2 | | 1.5 – 3.0 | | |
| Widowed | 52.5 | | | 49.7 – 55.3 | | | 44.2 | | | 41.5 – 47.0 | | | 3.3 | | 2.3 – 4.3 | | |
| Separated, divorced, single | 64.3 | | | 52.8 – 75.8 | | | 34.3 | | | 22.9 – 45.7 | | | 1.4 | | -1.4 – 4.3 | | |
| *Living with spouse* |  | | |  | | |  | | |  | | |  | |  | | |
| Yes | 66.7 | | | 63.5 – 69.8 | | | 30.6 | | | 27.5 – 33.7 | | | 2.7 | | 1.7 – 3.8 | | |
| No | 59.0 | | | 56.9 – 61.2 | | | 38.4 | | | 36.2 – 40.5 | | | 2.6 | | 1.9 – 3.3 | | |
| *Household head* | |  | | |  | | |  | | |  | |  | | |  | |
| Yes | | 66.6 | | | 64.3 – 69.0 | | | 31.1 | | | 28.8 – 33.5 | | 2.2 | | | 1.5 – 3.0 | |
| No | | 55.5 | | | 52.8 – 58.1 | | | 41.4 | | | 38.8 – 44.0 | | 3.1 | | | 2.2 – 4.1 | |
| *Working status* | |  | | |  | | |  | | |  | |  | | |  | |
| Yes | | 70.3 | | | 67.7 – 73.0 | | | 28.6 | | | 26.0 – 31.2 | | 1.0 | | | 0.5 – 1.6 | |
| No | | 55.2 | | | 52.8 – 57.5 | | | 41.1 | | | 38.8 – 43.4 | | 3.7 | | | 2.8 – 4.6 | |
| *Wealth quintiles* | |  | | |  | | |  | | |  | |  | | |  | |
| Richest | | 66.0 | | | 62.2 – 69.8 | | | 31.2 | | | 27.5 – 34.9 | | 2.8 | | | 1.5 – 4.1 | |
| Richer | | 62.9 | | | 59.2 – 66.7 | | | 35.1 | | | 31.3 – 38.8 | | 2.0 | | | 0.9 – 3.1 | |
| Middle | | 61.5 | | | 57.8 – 65.3 | | | 36.4 | | | 32.7 – 40.2 | | 2.0 | | | 0.9 – 3.1 | |
| Poorer | | 58.5 | | | 54.1 – 62.8 | | | 39.3 | | | 35.0 – 43.6 | | 2.2 | | | 0.9 – 3.5 | |
| Poorest | | 56.0 | | | 51.6 – 60.4 | | | 39.6 | | | 35.2 – 43.9 | | 4.4 | | | 2.6 – 6.2 | |
| *National poverty line* | |  | | |  | | |  | | |  | |  | | |  | |
| Above | | 61.7 | | | 59.7 – 63.6 | | | 35.7 | | | 33.8 – 37.6 | | 2.6 | | | 1.9 – 3.2 | |
| Beyond | | 59.1 | | | 54.4 – 63.8 | | | 37.9 | | | 33.2 – 42.5 | | 3.0 | | | 1.4 – 4.7 | |

**Table A5 – Percentage of older people among socioeconomic groups**

**by levels of anxiety/depression dimension**

| *Problems* |  | | *No* | | |  |  | | *Some/moderate* | | |  |  | *Severe* | | |  |
| --- | --- | --- | --- | --- | --- | --- | --- | --- | --- | --- | --- | --- | --- | --- | --- | --- | --- |
| *Socioeconomic groups* | *%* | | | *95%CI* | | | *%* | | | *95%CI* | | | *%* | | *95%CI* | | |
| *Age groups* |  | | |  | | |  | | |  | | |  | |  | | |
| 60–69 | 83.4 | | | 81.3 – 85.5 | | | 14.8 | | | 12.8 – 16.8 | | | 1.8 | | 1.1 – 2.6 | | |
| 70–79 | 81.8 | | | 79.5 – 84.2 | | | 16.0 | | | 13.8 – 18.2 | | | 2.1 | | 1.3 – 3.0 | | |
| 80–89 | 79.8 | | | 76.4 – 83.3 | | | 17.4 | | | 14.1 – 20.7 | | | 2.7 | | 1.3 – 4.2 | | |
| 90+ | 72.4 | | | 62.1 – 82.7 | | | 25.0 | | | 15.0 – 35.0 | | | 2.6 | | -1.1 – 6.3 | | |
| *Gender* |  | | |  | | |  | | |  | | |  | |  | | |
| Male | 85.4 | | | 83.2 – 87.5 | | | 13.6 | | | 11.5 – 15.7 | | | 1.0 | | 0.4 – 1.7 | | |
| Female | 79.8 | | | 78.0 – 81.7 | | | 17.4 | | | 15.6 – 19.1 | | | 2.8 | | 2.0 – 3.5 | | |
| *Education* |  | | |  | | |  | | |  | | |  | |  | | |
| High school and higher | 91.4 | | | 87.6 – 95.1 | | | 6.8 | | | 3.5 – 10.2 | | | 1.8 | | 0.0 – 3.6 | | |
| Primary/secondary school | 85.3 | | | 83.2 – 87.4 | | | 13.6 | | | 11.6 – 15.6 | | | 1.1 | | 0.5 – 1.7 | | |
| Read and write only | 79.3 | | | 76.8 – 81.8 | | | 18.0 | | | 15.6 – 20.4 | | | 2.7 | | 1.7 – 3.7 | | |
| Illiterate | 75.4 | | | 71.7 – 79.1 | | | 21.1 | | | 17.6 – 24.7 | | | 3.5 | | 1.9 – 5.1 | | |
| *Marital status* |  | | |  | | |  | | |  | | |  | |  | | |
| Married | 84.8 | | | 83.1 – 86.6 | | | 13.8 | | | 12.0 – 15.5 | | | 1.4 | | 0.8 – 2.0 | | |
| Widowed | 78.7 | | | 76.4 – 81.0 | | | 18.2 | | | 16.1 – 20.4 | | | 3.0 | | 2.1 – 4.0 | | |
| Separated, divorced, single | 69.6 | | | 58.4 – 80.7 | | | 27.5 | | | 16.7 – 38.3 | | | 2.9 | | -1.2 – 7.0 | | |
| *Living with spouse* |  | | |  | | |  | | |  | | |  | |  | | |
| Yes | 86.0 | | | 83.7 – 88.3 | | | 12.7 | | | 10.5 – 14.9 | | | 1.3 | | 0.5 – 2.0 | | |
| No | 80.0 | | | 78.3 – 81.8 | | | 17.5 | | | 15.8 – 19.1 | | | 2.5 | | 1.8 – 3.2 | | |
| *Household head* | |  | | |  | | |  | | |  | |  | | |  | |
| Yes | | 81.5 | | | 79.5 – 83.4 | | | 16.3 | | | 14.4 – 18.1 | | 2.3 | | | 1.5 – 3.0 | |
| No | | 82.4 | | | 80.4 – 84.4 | | | 15.6 | | | 13.7 – 17.6 | | 2.0 | | | 1.2 – 2.7 | |
| *Working status* | |  | | |  | | |  | | |  | |  | | |  | |
| Yes | | 83.9 | | | 81.8 – 86.0 | | | 14.5 | | | 12.5 – 16.6 | | 1.6 | | | 0.8 – 2.3 | |
| No | | 80.5 | | | 78.6 – 82.4 | | | 17.0 | | | 15.2 – 18.8 | | 2.5 | | | 1.8 – 3.3 | |
| *Wealth quintiles* | |  | | |  | | |  | | |  | |  | | |  | |
| Richest | | 87.9 | | | 85.3 – 90.5 | | | 10.8 | | | 8.3 – 13.3 | | 1.3 | | | 0.4 – 2.2 | |
| Richer | | 86.8 | | | 84.2 – 89.5 | | | 11.9 | | | 9.4 – 14.4 | | 1.3 | | | 0.4 – 2.1 | |
| Middle | | 81.1 | | | 78.1 – 84.2 | | | 17.0 | | | 14.1 – 19.9 | | 1.9 | | | 0.8 – 2.9 | |
| Poorer | | 80.7 | | | 77.2 – 84.2 | | | 16.9 | | | 13.5 – 20.2 | | 2.4 | | | 1.1 – 3.8 | |
| Poorest | | 70.4 | | | 66.3 – 74.4 | | | 25.4 | | | 21.6 – 29.2 | | 4.2 | | | 2.5 – 6.0 | |
| *National poverty line* | |  | | |  | | |  | | |  | |  | | |  | |
| Above | | 83.4 | | | 81.9 – 84.9 | | | 14.7 | | | 13.3 – 16.1 | | 1.9 | | | 1.4 – 2.5 | |
| Below | | 73.2 | | | 68.9 – 77.4 | | | 23.5 | | | 19.5 – 27.6 | | 3.3 | | | 1.6 – 5.0 | |
